# Supplementary material for: From sequence to enzyme mechanism using multi-label machine learning
Source: BMC Bioinformatics. 2014 May 19;15:150. doi: 10.1186/1471-2105-15-150 (PMC4229970; doi:10.1186/1471-2105-15-150)
Supplement: Additional file 2 — Java code of ml2db. Additional file ml2db_code.tar.gz contains the Java source code to run the multi-label machine learning experiments and save the results to database. The code’s Javadoc is included. [file 1471-2105-15-150-S2.zip › additional file 2/ml2db/ml2db/doc/mulan/classifier/lazy/MultiLabelKNN.html]

MultiLabelKNN


JavaScript is disabled on your browser.


- Overview
- Package
- Class
- Use
- Tree
- Deprecated
- Index
- Help

- Prev Class
- Next Class

- Frames
- No Frames

- All Classes

- Summary:
- Nested |
- Field |
- Constr |
- Method

- Detail:
- Field |
- Constr |
- Method


mulan.classifier.lazy

## Class MultiLabelKNN

- java.lang.Object
- - mulan.classifier.MultiLabelLearnerBase
  - - mulan.classifier.lazy.MultiLabelKNN

- All Implemented Interfaces:
  :   java.io.Serializable, MultiLabelLearner, weka.core.TechnicalInformationHandler

  Direct Known Subclasses:
  :   BRkNN, IBLR\_ML, MLkNN

  ---

    

  ```
  public abstract class MultiLabelKNN
  extends MultiLabelLearnerBase
  ```

  Superclass of all KNN based multi-label algorithms

  Author:
  :   Eleftherios Spyromitros-Xioufis, Grigorios Tsoumakas

  See Also:
  :   Serialized Form

- - ### Field Summary

    Fields

    | Modifier and Type | Field and Description |
    | --- | --- |
    | `static int` | `WEIGHT_INVERSE` weight by 1/distance. |
    | `static int` | `WEIGHT_NONE` no weighting. |
    | `static int` | `WEIGHT_SIMILARITY` weight by 1-distance. |
  - ### Constructor Summary

    Constructors

    Constructor and Description

    `MultiLabelKNN()`

    The default constructor

    `MultiLabelKNN(int numOfNeighbors)`

    Initializes the number of neighbors
  - ### Method Summary

    Methods

    | Modifier and Type | Method and Description |
    | --- | --- |
    | `boolean` | `isUpdatable()` Returns value indicating if learner is updatable, so if learner is able to perform on-line learning. |
    | `void` | `setDfunc(weka.core.DistanceFunction dfunc)` Sets a distance function |
    | `void` | `setDistanceWeighting(int distanceWeighting)` |

    - ### Methods inherited from class mulan.classifier.MultiLabelLearnerBase

      `build, getDebug, getTechnicalInformation, globalInfo, makeCopy, makePrediction, setDebug`
    - ### Methods inherited from class java.lang.Object

      `equals, getClass, hashCode, notify, notifyAll, toString, wait, wait, wait`

- - ### Field Detail


    - #### WEIGHT\_NONE

      ```
      public static final int WEIGHT_NONE
      ```

      no weighting.

      See Also:
      :   Constant Field Values


    - #### WEIGHT\_INVERSE

      ```
      public static final int WEIGHT_INVERSE
      ```

      weight by 1/distance.

      See Also:
      :   Constant Field Values


    - #### WEIGHT\_SIMILARITY

      ```
      public static final int WEIGHT_SIMILARITY
      ```

      weight by 1-distance.

      See Also:
      :   Constant Field Values
  - ### Constructor Detail


    - #### MultiLabelKNN

      ```
      public MultiLabelKNN()
      ```

      The default constructor


    - #### MultiLabelKNN

      ```
      public MultiLabelKNN(int numOfNeighbors)
      ```

      Initializes the number of neighbors

      Parameters:
      :   `numOfNeighbors` - the number of neighbors
  - ### Method Detail


    - #### setDfunc

      ```
      public void setDfunc(weka.core.DistanceFunction dfunc)
      ```

      Sets a distance function

      Parameters:
      :   `dfunc` - the distance function


    - #### isUpdatable

      ```
      public boolean isUpdatable()
      ```

      **Description copied from interface: `MultiLabelLearner`**

      Returns value indicating if learner is updatable, so if learner is able to
      perform on-line learning. The fact if learner is updatable or not influences
      the behavior of `MultiLabelLearner.build(MultiLabelInstances)` method.  
      When `false` is returned, each call of the
      `MultiLabelLearner.build(MultiLabelInstances)` will initialize the learner from
      the scratch, removing any potential knowledge built by previously entered training data.
      When `true` is returned, then on the first call of the
      `MultiLabelLearner.build(MultiLabelInstances)` the learner is initialized
      with the passed training data. All other calls contribute to the existing learner's
      model with new data.

      **Specified by:**
      :   `isUpdatable` in interface `MultiLabelLearner`

      **Overrides:**
      :   `isUpdatable` in class `MultiLabelLearnerBase`

      Returns:
      :   `true` if learner is updatable (on-line), `false` otherwise.


    - #### setDistanceWeighting

      ```
      public void setDistanceWeighting(int distanceWeighting)
      ```

      Parameters:
      :   `distanceWeighting` - the distanceWeighting to set


- Overview
- Package
- Class
- Use
- Tree
- Deprecated
- Index
- Help

- Prev Class
- Next Class

- Frames
- No Frames

- All Classes

- Summary:
- Nested |
- Field |
- Constr |
- Method

- Detail:
- Field |
- Constr |
- Method
